# Long-Noncoding RNA MANCR is Associated With Head and Neck Squamous Cell Carcinoma Malignant Development and Immune Infiltration

**DOI:** 10.3389/fgene.2022.911733

**Published:** 2022-07-08

**Authors:** Jianfei Tang, Mingyan Bao, Juan Chen, Xin Bin, Xinghuanyu Xu, Xiaodan Fang, Zhangui Tang

**Affiliations:** Hunan Key Laboratory of Oral Health Research and Hunan 3D Printing Engineering Research Center of Oral Care and Hunan Clinical Research Center of Oral Major Diseases and Oral Health and Academician Workstation for Oral-maxilofacial and Regenerative Medicine and Xiangya Stomatological Hospital and Xiangya School of Stomatology, Central South University, Changsha, China

**Keywords:** MANCR, LncRNA, HNSCC, immune infiltration, bioinformatics analysis, cancer

## Abstract

Recent studies have demonstrated an important role for mitotically associated long non-coding RNA (MANCR) in carcinogenesis and cancer progression, but its function has not been elucidated in head and neck squamous cell carcinoma (HNSCC). In this study, we identified differentially expressed MANCR from The Cancer Genome Atlas (TCGA) and Genotype-Tissue Expression (GTEx) databases across 24 cancer types and included 546 HNSCC patients. Furthermore, high expression of MANCR was verified in HNSCC cell lines and tissue by using real-time quantitative PCR (RT-qPCR) analysis. The Kaplan–Meier analysis showed a worse prognosis with higher levels of MANCR for HNSCC. The univariate Cox regression and multivariate Cox regression analyses revealed that MANCR was a high-risk factor in patients with HNSCC. Thereafter, we carried out the Gene Ontology (GO) and Kyoto Encyclopedia of Genes and Genomes (KEGG) enrichment analyses. It was indicated that MANCR participates in axonogenesis and ECM-receptor interaction. Further enrichment analysis demonstrated that the expression of MANCR was positively correlated with the T gamma delta (tgd) cells, neutrophils, and Th1 cells, and negatively correlated with the infiltration of B cells, CD8 T cells, and T cells in HNSCC. In addition, *in vitro* experiments showed that knockdown of MANCR in HNSCC cells markedly inhibited cell proliferation, migration, and invasion. We find that MANCR was elevated in HNSCC and promoted the malignant progression of HNSCC. MANCR may serve as a potential biomarker in prognostic implications for HNSCC patients. The positive correlation between MANCR and immune infiltration cells may provide novel therapeutic targets and personalized immune-based cancer therapy for HNSCC.

## Introduction

Head and neck squamous cell carcinoma (HNSCC) is the sixth most common malignancy worldwide, and seriously endangers human health (Johnson et al., 2020). The global incidence of HNSCC continues to rise. According to the Global Cancer Statistics 2020, about 878,348 new cases were diagnosed and 444,347 deaths occurred due to HNSCC (Sung et al., 2021). At present, surgery combined with adjuvant radiation and chemotherapy is the main and effective treatment method for HNSCC (Cramer et al., 2019). Even though advancements in treatment strategies were made in recent years, the five-year overall survival rate is only around 50% (Szturz et al., 2020). The poor prognosis in HNSCC can be mainly attributed to late-stage diagnosis, local recurrence, and distant metastasis. Therefore, it is important to figure out new biomarkers for clinical application, which may benefit the development of the diagnosis and therapy of HNSCC.

Long non-coding RNAs (lncRNAs) are defined as a type of broad transcripts that do not encode proteins or peptides with a length longer than 200 nucleotides (Quinn and Chang, 2016; Statello et al., 2021). Presently, numerous studies revealed the important functions of lncRNAs in diseases, especially cancers (Bhan et al., 2017). Mitotically associated long non-coding RNA (MANCR) is a newly identified lncRNA which was reported to be involved in various cancers. Upregulation of MANCR is significantly related to the poor prognosis of breast cancer *via* affecting the stability of the genome, leading to DNA damage and cell division defects (Tracy et al., 2018). In hepatocellular carcinoma, MANCR could promote the tumor cell proliferation process by regulating miR-122a (Zhang et al., 2019). The downregulation of MANCR could inhibit mantle cell lymphoma proliferation by interacting with RUNX2 (Wen et al., 2019). A study reported that the upregulation of MANCR has the ability to enhance EMT-related functions. For example, BET inhibitor JQ1 could directly act on MANCR to inhibit the migration and invasion of prostate cancer cells (Nagasawa et al., 2020). The dysregulated expression levels of MANCR were reported in thyroid carcinoma, gastric carcinoma, and esophagus cancer (Yao et al., 2019; Huang et al., 2020; Fan and Wang, 2021), thus enhancing tumor malignant development. However, with regard to HNSCC, few studies have focused on the potential function of MANCR in diagnosis and prognosis. As far as we know, there is no research on systematically evaluating the function of MANCR in HNSCC by the bioinformatics approach. Hence, we used lncRNA expression profiles of HNSCC from our previous study, combined with bioinformatics analysis and experiments for investigating the expression, clinical significance, and biological behavior influence of MANCR in HNSCC.

## Materials and Methods

### Data Collection and Bioinformatics Analysis

The lncRNA expression profiles and data were obtained from our previous study (Fang et al., 2020). A total of 546 HNSCC cases and their information were downloaded from The Cancer Genome Atlas (TCGA) database (http://cancergenome.nih.gov) and the GEO database (GSE97251). Genotype-Tissue Expression (GTEx) clinical pan-cancer data were downloaded from an online website (https://gtexp.ortal.org/home/datasets). A Venn analysis was used by the R software package (v3.3.3). Related clinicopathological data were obtained from TCGA, including TN stage, clinical stage, histologic grade, smoking, and alcohol history.

### Survival Prognosis Analysis

We used Xiantao tools to analyze disease-specific survival (DSS) and overall survival (OS) of MANCR in HNSCC patients. A receiver operating characteristic (ROC) curve analysis was used to evaluate diagnostic potential. Subgroup analyses were used to predicate the OS of HNSCC patients related to MANCR expression and clinical information. Univariate and multivariate Cox regression analyses were used to explore potential risk factors in patients with HNSCC by different characteristics such as T and N stages, clinical stage, histologic grade, age, gender, radiation therapy, race, lymphovascular invasion, lymph node neck dissection, primary therapy outcome, and MANCR expression, with the hazard ratio (HR), 95% confidence interval (CI), and log-rank *p*-value. All the survival plots were visualized by the survminer package (version 0.4.9). Based on the potential value characteristics of multivariate Cox regression, we selected these characteristics to construct a nomogram for predicting the OS of HNSCC patients, and the accuracy of the prediction model was evaluated by the matched degree of the calibration curve and line of 1-, 3-, and 5-year OS.

### Clinical Specimen Collection

All the 49 pairs of HNSCC and adjacent normal tissues were collected from patients who had undergone surgical resection at Xiangya Stomatological Hospital, Central South University (Changsha, China), and were enrolled in this study for RT-qPCR analysis. The extracted adjacent normal tissues were at least ≥2 cm away from the primary tumor. All patients did not undergo surgery, radiotherapy, or chemotherapy before. All samples were evaluated by two different pathologists for a clear pathological diagnosis, and tissues are stored at −80°C for total RNA extraction. All samples were obtained with written informed consent, and the operation was approved by the Ethics Committee of Xiangya Stomatological Hospital of Central South University.

### RNA Extraction and RT-qPCR Analysis

According to the manufacturer’s instructions for the TRIzol reagent (Invitrogen, United States) and cDNA synthesis kit (Vazyme, China), the total RNA of tissues was extracted and reversely transcribed into cDNA. Following the manufacturer’s instructions of the ChamQ Universal SYBR qPCR Master Mix (Vazyme, China), PCR amplification was performed to detect the lncRNA expression level by using the real-time PCR system (Applied Biosystems, United States). GAPDH was used as an endogenous control gene for normalization. Relative primer sequences were synthesized by Sangon Biotech (Shanghai, China), and all the primer sequences were as follows: GAPDH forward primer:5′-CTGCCAACGTGTCAGTGGTG-3’; reverse primer: 5′-TCA​GTG​TAG​CCC​AGG​ATG​CC-3’; MANCR forward primer: 5′-GCA​GAC​AGA​TTC​AGC​ACC​AGG​AG-3’; reverse primer: 5′-CCA​CCA​TGC​CAG​GCC​GAA​AC-3’. The 2^−ΔΔ^CT method was performed to calculate differences in MANCR expression levels between samples.

### Cell Culture and Transfection

The HNSCC cell lines (Cal27, SCC9, and HN30) were cultured in Dulbecco’s Modified Eagle Medium (DMEM; BI, Spain) containing 10% fetal bovine serum (FBS; BI, Spain), 100 U/mL penicillin, and 100 μg/ml streptomycin; the human oral keratinocyte cell (Hok) was cultured in Minimum Essential Medium α (MEM-α, BI, Spain) containing 10% FBS, 100 U/mL penicillin, and 100 μg/ml streptomycin, and both of these cells were maintained in a humidified incubator at 37°C, 5% CO2 conditions.

The lncRNA Smart Silencer targeting MANCR (si-MANCR) and negative controls (NC) were obtained from RiboBio Corporation (Guangzhou, China). Smart Silencer sequences comprise three antisense oligonucleotides (ASOs) and three siRNAs. The targeting sequences of si-MANCR were as follows: ASO sequences, 5′-CAA​TCA​AAA​GAC​GGC​TTT​A-3’; 5′-CTC​AAT​CAC​CAC​AAT​TGC​A-3’; 5’ -TCA​CCA​CAA​TTG​CAA​TCA​A-3’; siRNA sequences, 5′-AAA​TGG​CAA​GTT​TCG​GCC​TG-3’; 5’ -ACA​TCC​ACT​CAC​CAC​TCG​CT-3’; 5′-CTC​CTT​TCT​TAC​ATA​TCC​AC-3’. The transfection reagent mixture was prepared according to the manufacturer’s instructions of the riboFECT CP Transfection Kit: 120 μl 1×riboFECTCP Buffer, 5 μl 20 μm Smart Silencer solution, and 12 μL riboFECT CP Reagent. Cells grown at 30%–50% confluence were transfected with si-MANCR and NC mixture, respectively, for 48 h. After 48h, the transfected cells will be collected for efficiency detecting by RT-qPCR and for further experiments.

### Cell Proliferation Assay

After 48 h of transfection, Cal27 and SCC9 cells were seeded in 96-well-plates (1 × 10^4^ cell/well) for cell viability detection by using a Cell Counting Kit-8 (CCK-8, Dojindo, Japan). Transfected cells were incubated for 0, 24, 48, and 72 h. The mixture of 10 μl CCK8 solution and 100 μl medium was added to each well and cultured for another 2 h at 37°C at different time points (24 h, 48 h, and 72 h). The OD value at 450 nm was measured by using microplate reader (BioTeK, United States).

### Migration and Invasion Assay

The migrative and invasive capacity of SCC9 and Cal27 cells was assayed by using a 24-well transwell chamber (BD Biosciences, United States). The transfected SCC9 or Cal27 cells were harvested and suspended in serum-free DMEM, and then 100 μl cell suspension (1×10^5^ cells) was added into the upper chamber with pre-coated Matrigel (BD Biosciences, United States) for the invasion assay, whereas for the migration assay, the upper chamber should contain uncoated-Matrigel, then 600 μl DMEM with 20% FBS was added into the lower chamber. After 48 h of incubation, the residual medium in the upper chamber should be tenderly removed. The invasive and migrative cells in the lower membrane surface were fixed with 4% paraformaldehyde and stained with 0.1% crystal violet. After washing with PBS, the number of stained cells on the lower membrane surface was counted in five randomly selected fields under a light microscope (Leica, German) and the photos were analyzed by ImageJ software (version.1.8.0).

### Function and Gene Set Enrichment Analysis

The TCGA HNSC data were used for Pearson correlation analysis of differently expressed genes (DEGs) which were related to MANCR. |log^2^(FC)| > 2 and *p* < 0.05 were regarded as the cut-off criteria and the expression was visualized by the R software package (ggplot2, v3.3.3). Gene Ontology (GO) and Kyoto Encyclopedia of Genes and Genomes (KEGG) pathways with the R software package (clusterProfiler) were used to analyze the biological process (BP), cellular component (CC), molecular function (MF), and related potential signaling. The cut-off criterion is *P* < 0.05 and enrichment score > 1.5. GSEA is a practical tool used to assess the trend in the distribution of genes of a predefined gene set. Then, we used the GSEA analysis to explore potential pathways in high- and low-MANCR groups of DEGs by applying the R software package clusterProfiler (version 3.6.3). In detail, the enriched standard was *p*-value (q-value) < 0.25, adjusted *p* < 0.05, and |normalized enrichment score| > 1.

### Immune Cell Infiltration Analysis

To assess immune cells infiltration scores in HNSCC from TCGA database, we performed the single-sample gene enrichment analysis (ssGSEA). We used the GSVA R package to compare normalized MANCR expression level data and different immune cell signatures, and the specific enrichment scores of 24 immune cells were calculated and exhibited. Furthermore, the correlations between MANCR and immune cell infiltration levels were revealed by Spearman’s rank correlation test and Wilcoxon’s rank-sum test (*P* < 0.05).

### Statistical Analysis

All data were shown as mean ± standard deviation (SD). GraphPad Prism 9.0 was used to analyze the experimental result. The clinical implication of MANCR was analyzed by the Wilcoxon rank sum test. A prognostic analysis was performed by the Kaplan–Meier survival analysis and Cox univariate and multivariate analyses. Student’s t-test was used to evaluate the pairwise differences between the groups. *p* < 0.05 was considered a statistically significant difference. The R software package (ggplot2, v3.3.3) was from Xiantao tools (https://www.xiantao.love/).

## Results

### Expression of MANCR in Various Cancers Including HNSCC

**FIGURE 1:**
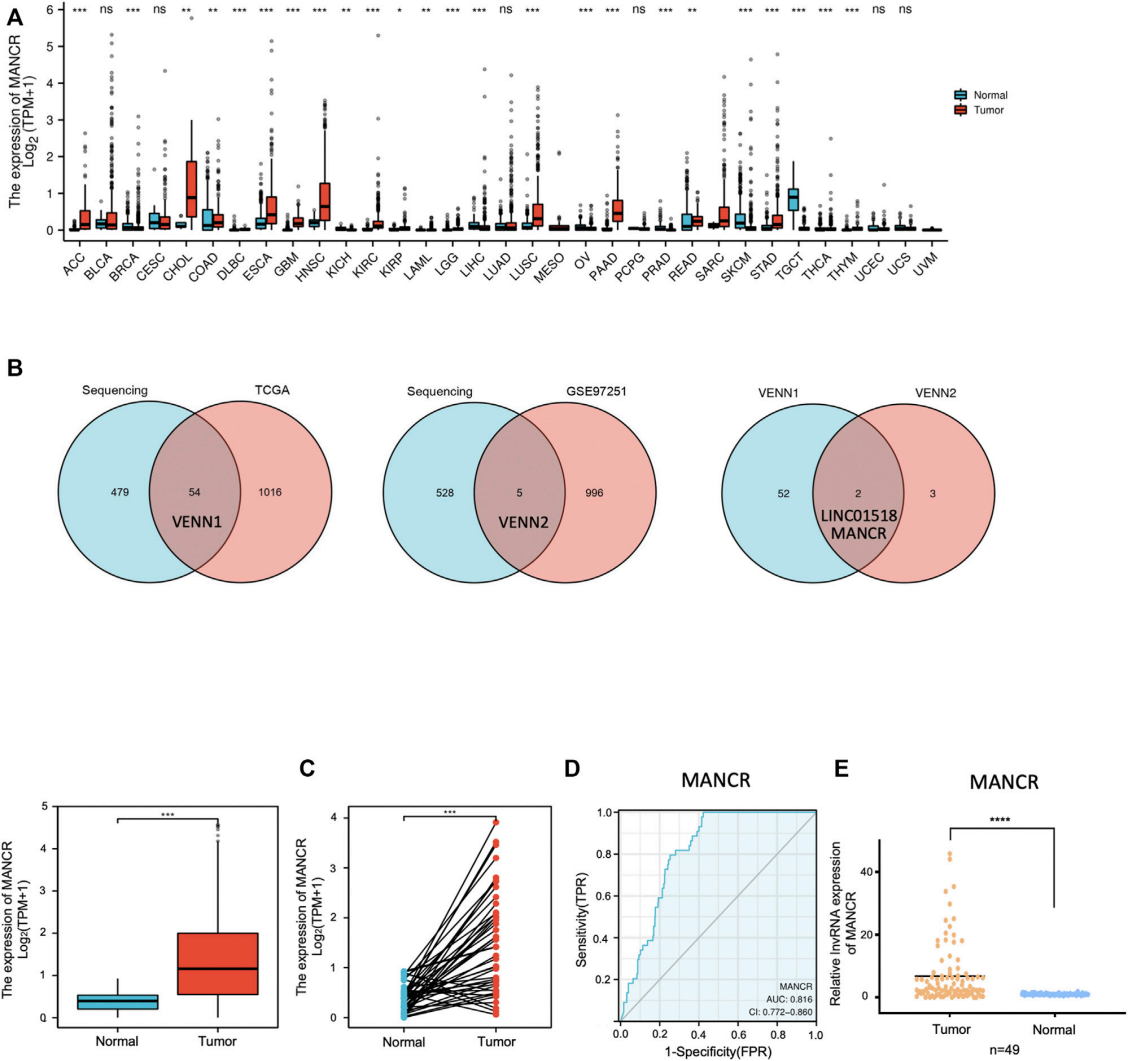
MANCR expression level in pan-cancers including HNSCC. **(A)** Different expression levels of MANCR in most cancer types from TGCA and GTEx data (*p < 0.05, **p < 0.01, ***p < 0.001, and ****p < 0.0001). **(B)** Venn analysis of previous lncRNA expression profiles, TCGA, and GEO databases. **(C)** MANCR expression of HNSCC tumor samples and adjacent normal samples from TCGA data (***p < 0.001). **(D)** ROC curve for MANCR expression in TCGA HNSCC. **(E)** RT-qPCR result of MANCR expression in 49 HNSCC tumor tissues and adjacent normal tissues. Data were shown as mean ± SD (*p < 0.05, **p < 0.01, ***p < 0.001, and ****p < 0.0001).

We first used TCGA and the GTEx pan-cancer database to evaluate the expression level of MANCR in various cancer types. The result showed that MANCR expression was elevated in 17 cancers, namely, BLCA, COAD, DLBC, ESCA, GBM, HNSC, KICH, KIRP, LAML, LIHC, OV, PAAD, PCPG, SKCM, TGCT, YHYM, and UCEC, and downregulated in seven cancers, namely, CESC, KIRC, LGG, LUAD, READ, STAD, and THCA (Figure 1A). Then, we further confirmed the high expression level of MANCR in HNSCC by the Venn analysis (Figure 1B). Particularly, high MANCR expression was observed in HNSCC tissues compared with normal tissues from TCGA database (Figure 1C). The ROC curve was generated to assess the ability of MANCR to differentiate HNSCC from normal tissues. The AUC area was 0.816 (95% CI = 0.772–0.860) (Figure 1D). We also used RT-qPCR to validate the expression levels of MANCR in HNSCC tissues. The result showed that MANCR was highly expressed in HNSCC samples compared with the adjacent tissues (Figure 1E). Moreover, the high expression of MANCR was significantly associated with the N stage (*p* = 0.006) (Table 1).

**TABLE 1:** Information on HNSCC patients and association with MANCR expression.

Characteristics	High (*n* = 40)	Low (*n* = 9)	*p-*value
Gender			
Male	33	9	0.322
Female	7	0	
Age			
≤60	20	8	0.059
>60	20	1	
T Stage			
T1–T2	23	8	0.127
T3–T4	17	1	
N Stage			
N0	20	9	0.006**
N1–N3	20	0	
Location			
Lips	1	0	0.664
Gingiva	5	0	
Tongue	18	7	
Buccal	10	1	
Oropharynx	2	1	
Maxilla	2	0	
Mouth floor	2	0	

*p*-values are based on chi-squared or Fisher’s exact test. P < 0.05 indicates a significant association among the variables (**P < 0.01).

### Association Between MANCR Expression and Prognosis

**FIGURE 2:**
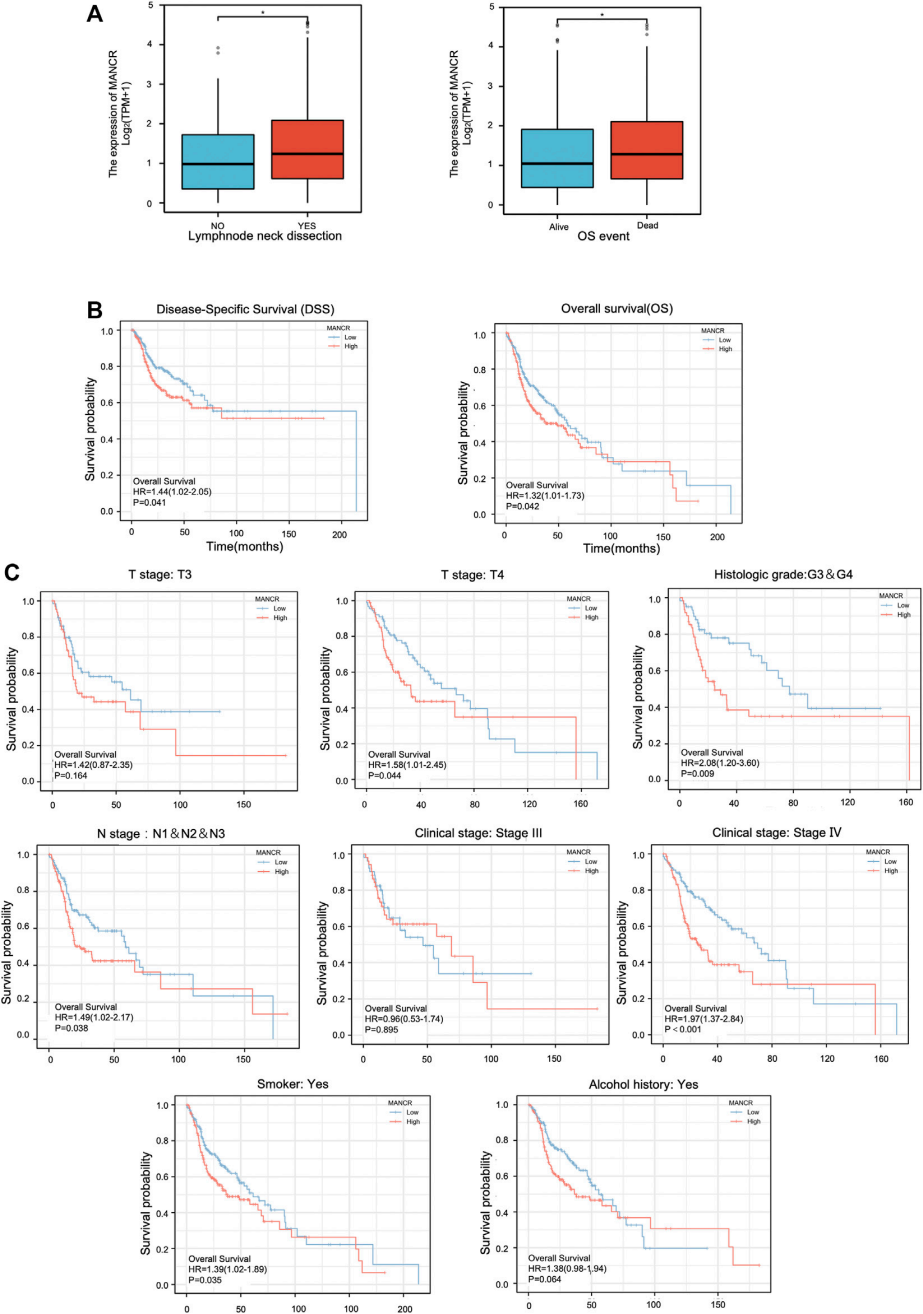
MANCR expression and prognosis correlation in HNSCC: **(A)** high expression level of MANCR was associated with lymph node neck dissection and poor OS. **(B)** Kaplan–Meier curves showed the overall survival and disease-specific survival in high- and low- MANCR expression of patient groups. **(C)** Subgroup analysis including the TN stage, clinical stage, histologic grade, smoking, and alcohol history. Data were shown as mean ± SD (*p < 0.05, **p < 0.01, ***p < 0.001, and ****p < 0.0001).

According to TCGA database of 546 HNSCC patients, the clinical correlation of MANCR expression was analyzed. The Wilcoxon rank sum text showed that MANCR expression was elevated in both HNSCC patients who underwent lymph node neck dissection and poor OS (Figure 2A). Furthermore, the Kaplan–Meier curve and log-rank test was used to evaluate the prognosis and clinicopathological parameters of MANCR expression in HNSCC patients. The results suggested that upregulation of MANCR was significantly associated with a poor OS (*p* = 0.042) and DSS (*p* = 0.041) in patients with HNSCC (Figure 2B). In addition, the subgroup analysis exhibited the correlation between MANCR expression and the OS of HNSCC patients with different characteristics (Figure 2C). In patients with lymph metastasis, with histologic grades Ⅳ, at the T4 stage, with clinical stages Ⅳ, and smoking patients, high MANCR expression predicated poor prognosis.

### Prognostic Value of MANCR Expression in HNSCC

**FIGURE 3:**
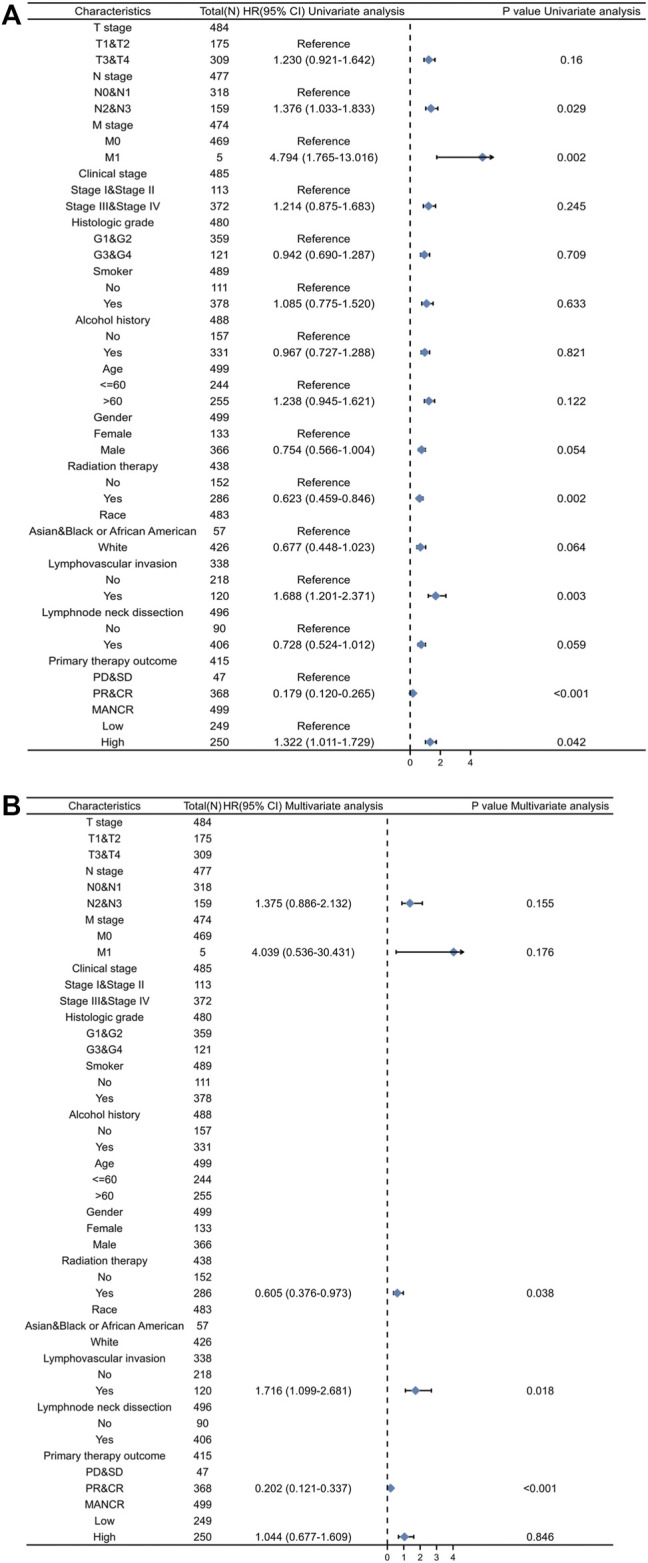
Univariate **(A)** and multivariate **(B)** Cox regression analyses of clinicopathological parameters and overall survival (*p* < 0.05 was regarded as significant).

**FIGURE 4:**
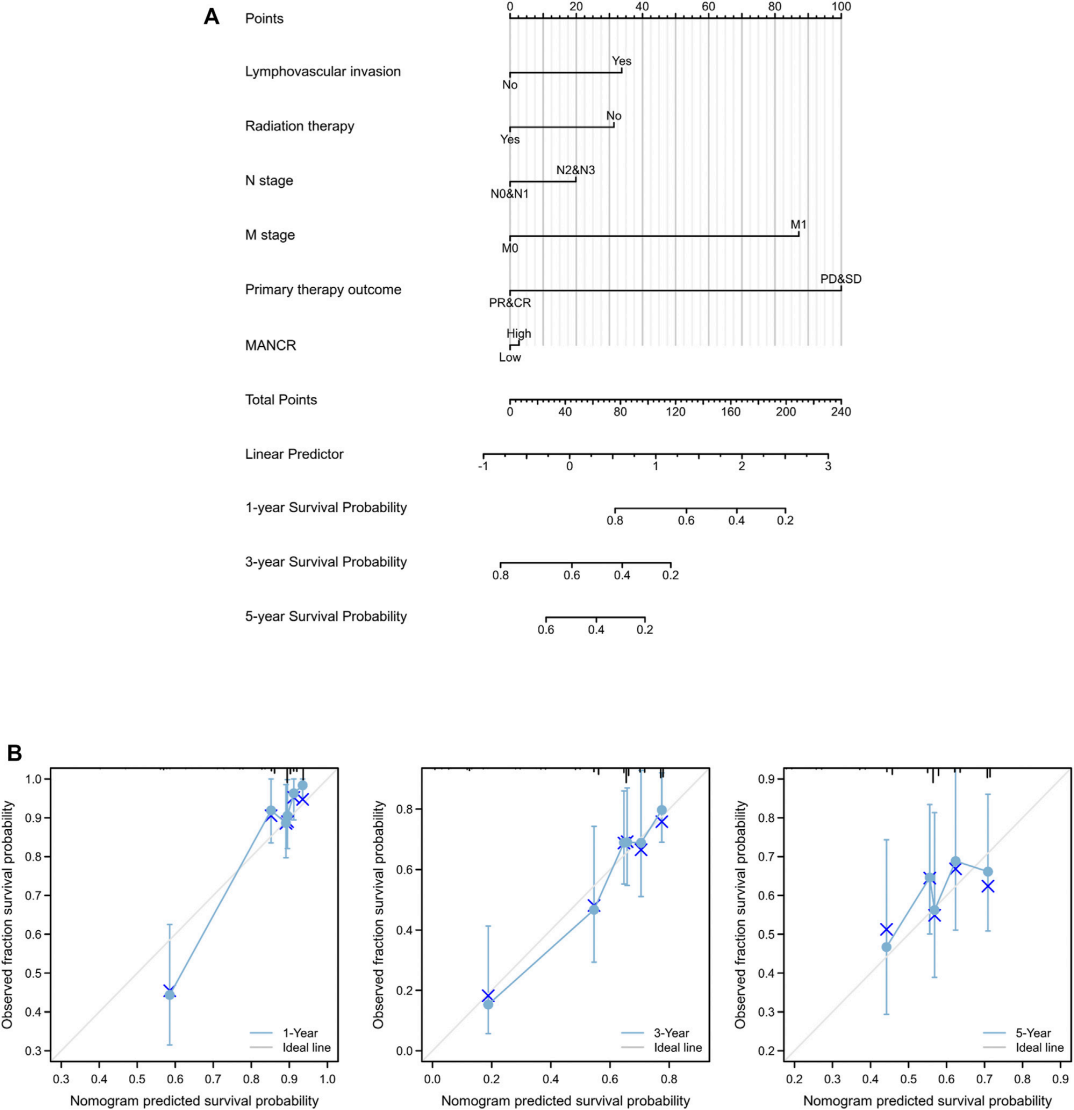
Role of MANCR in predicting the prognosis of HNSCC patients. **(A)** Nomogram. **(B)** Calibration curve at different time points.

Next, we evaluated the potential prognostic value of MANCR by univariate and multivariate Cox regression. Multivariate Cox regression of the OS indicated that radiation therapy, lymph vascular invasion, and primary therapy outcome were independent risk factors in patients with HNSCC, while the upregulation of MANCR did not exhibit statistical significance (Figure 3B). In particular, univariate Cox regression of OS suggested that high MANCR expression level was a high-risk factor in patients with HNSCC (*p* = 0.042) and it was associated with shorter survival (HR = 1.044) (Figure 3A). Therefore, we constructed a prognostic nomogram by the levels of expression of MANCR, radiation therapy, primary therapy outcome, N stage, M stage, and lymph vascular invasion to further predict individual survival probability (Figure 4A). The calibration curve of our model indicated that the established lines of survival at different time points (1, 3, and 5 years) highly matched with the ideal line (Figure 4B). The concordance (C-index) of our prognostic nomogram reached 0.716 (0.688–0.743), suggesting that the model had a reliable potential to predict OS.

### Correlation and Enrichment Analyses

**FIGURE 5:**
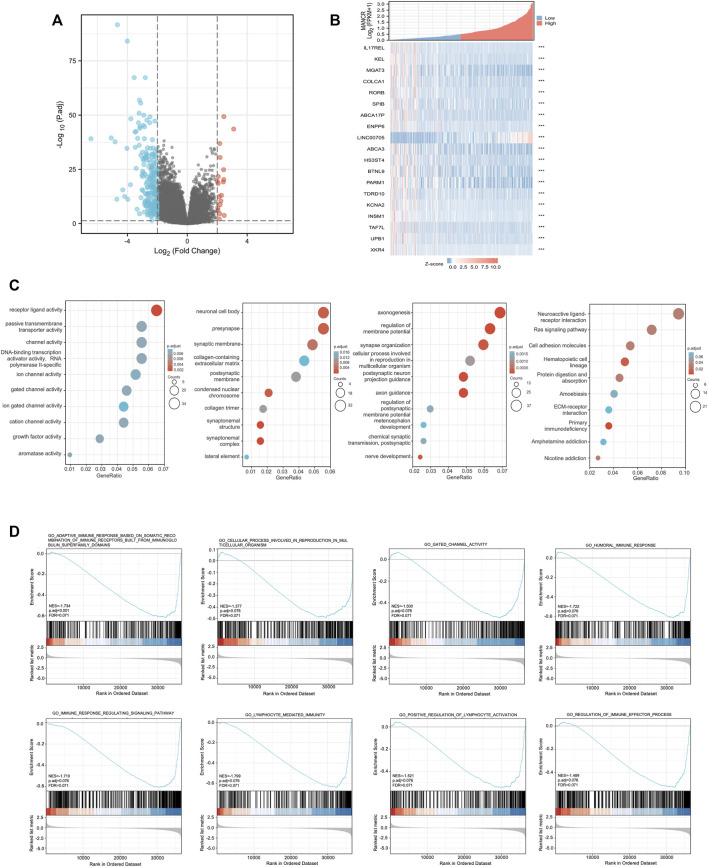
Differentially expressed genes (DEGs) in different MANCR expression samples and functional cluster analysis. **(A)** Volcano map of DEGs showed that 19 genes were upregulated and 205 genes were downregulated respectively (|log2FC| >2, adjusted *p-*value < 0.05). **(B)** Heat map showed that 19 genes were most significantly related to MANCR expression. **(C)** Significantly enriched GO and KEGG annotations of MANCR-related genes. **(D)** Enrichment gene analysis by GSEA in HNSCC.

To further explore the potential function and mechanism of MANCR in HNSCC development, we identified 224 differentially expressed genes (DEGs) in total by correlation analysis from TCGA database (|log^2^(FC)| > 2, *p* < 0.05) (Figure 5A). In particular, as the heat map showed, 19 genes were most significantly related to MANCR expression (Figure 5B). Next, we used GO and KEGG enrichment analyses to reveal the top 10 GO and KEGG terms in MANCR (Figure 5C). With regard to the GO analysis, MANCR was significantly and mainly associated with axonogenesis (BP), neuronal cell body (CC), and receptor-ligand activity (MF). The KEGG analysis suggested that neuroactive ligand-receptor interaction, Ras signaling pathway, and cell adhesion molecules were markedly enriched. In addition, we performed significant GSEA to explore potential pathways related to MANCR. The result showed that the most differentially enriched pathway is the activation of lymphocyte-mediated immunity. Other significant enriched pathways also included the activation of adaptive immune response based on somatic recombination of immune receptors built from immunoglobin superfamily domains, cellular processes involved in reproduction in multicellular organisms, gated channel activity, humoral immune response, immune response-regulating signaling pathway, positive regulation of lymphocyte activation, and regulation of immune effector processes (Figure 5D).

### Correlation Between Immune Cell Infiltration and MANCR Expression

**FIGURE 6:**
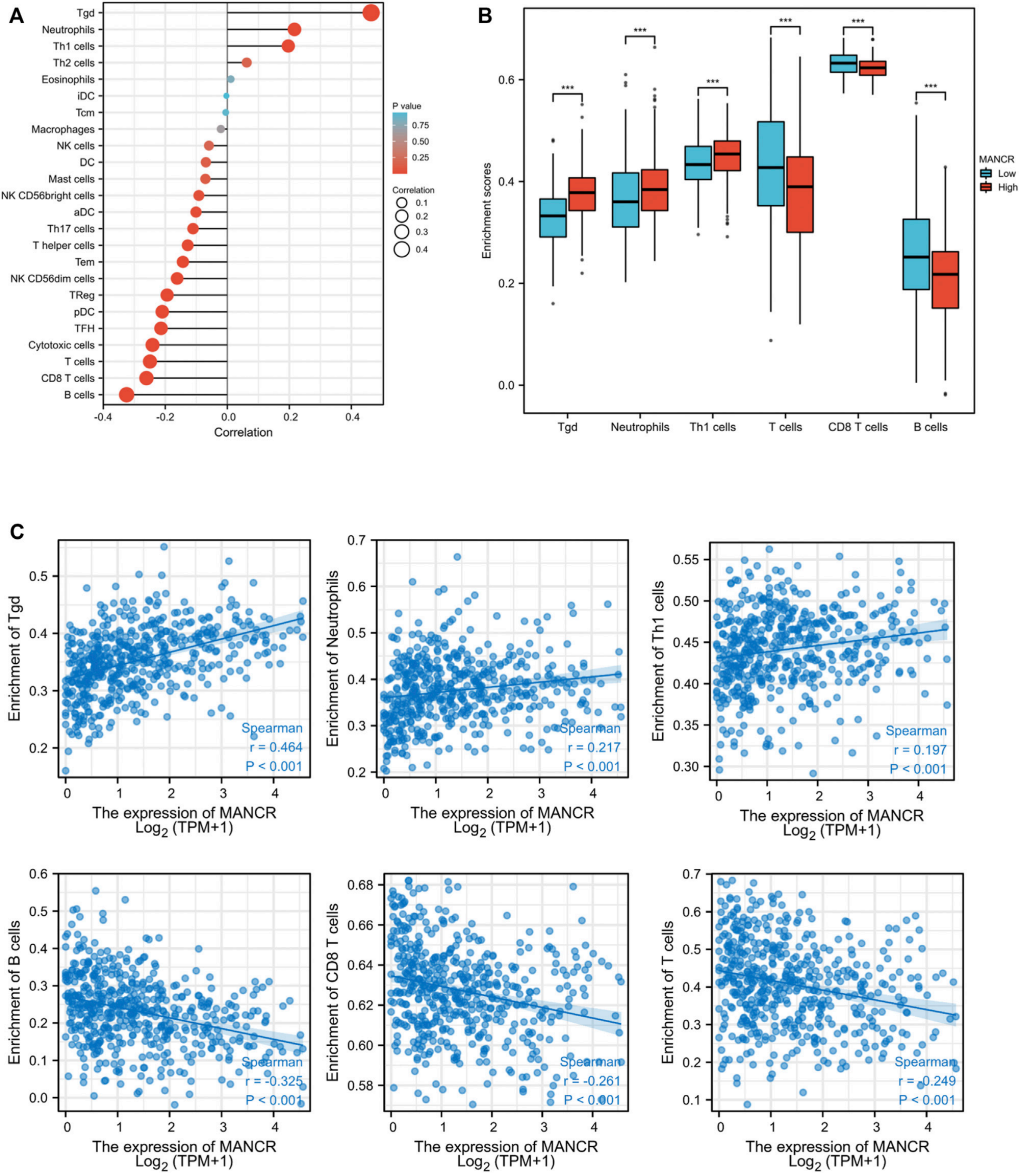
Correlation of MANCR expression with immune cell infiltration in HNSCC. **(A)** Lollipop indicates the correlation between MANCR expression and immune cells subsets. **(B)** Enrichment scores of tgd cells, neutrophils, Th1 cells, T cells, CD8 T cells, and B cell immune cells with MANCR expression. **(C)** Spearman analysis revealed the expression distribution of tgd cells, neutrophils, Th1 cells, T cells, CD8 T cells, and B cells in low and high MANCR samples. Data were shown as mean ± SD (*p < 0.05, **p < 0.01, ***p < 0.001, and ****p < 0.0001).

Based on the immune correlation from the results of the GSEA analysis, we explored the correlation between MANCR expression and immune cell infiltration by the Spearman and Pearson analysis. According to TCGA database and the ssGSEA algorithm, we find that MANCR expression was markedly positively correlated with T gamma delta (tgd) cells, neutrophils, and Th1 cell infiltration, whereas significantly negatively correlated with B cells, CD8 T cells, and T cell infiltration (Figure 6A). The independent sample t-test showed enrichment scores of those immune cells’ infiltration in the MANCR expression cohort (Figure 6B), and the Wilcoxon rank-sum test confirmed that tgd cells (Spearman r value = 0.464, *P* < 0.001), neutrophils (Spearman r value = 0.217, *P* < 0.001), and Th1 cells’ (Spearman r value = 0.197, *P* < 0.001) infiltration levels were higher in the MANCR-high expression group, while B cells (Spearman r value = −0.325, *P* < 0.001), CD8 T cells (Spearman r value = −0.261, *P* < 0.001), and T cells’ (Spearman r value = −0.249, *P* < 0.001) infiltration levels were higher in the MANCR-low expression group (Spearman r value = −0.325, *P* < 0.001) (Figure 5C).

### MANCR Knockdown Inhibited Malignant Behavior in HNSCC Cells

**FIGURE 7:**
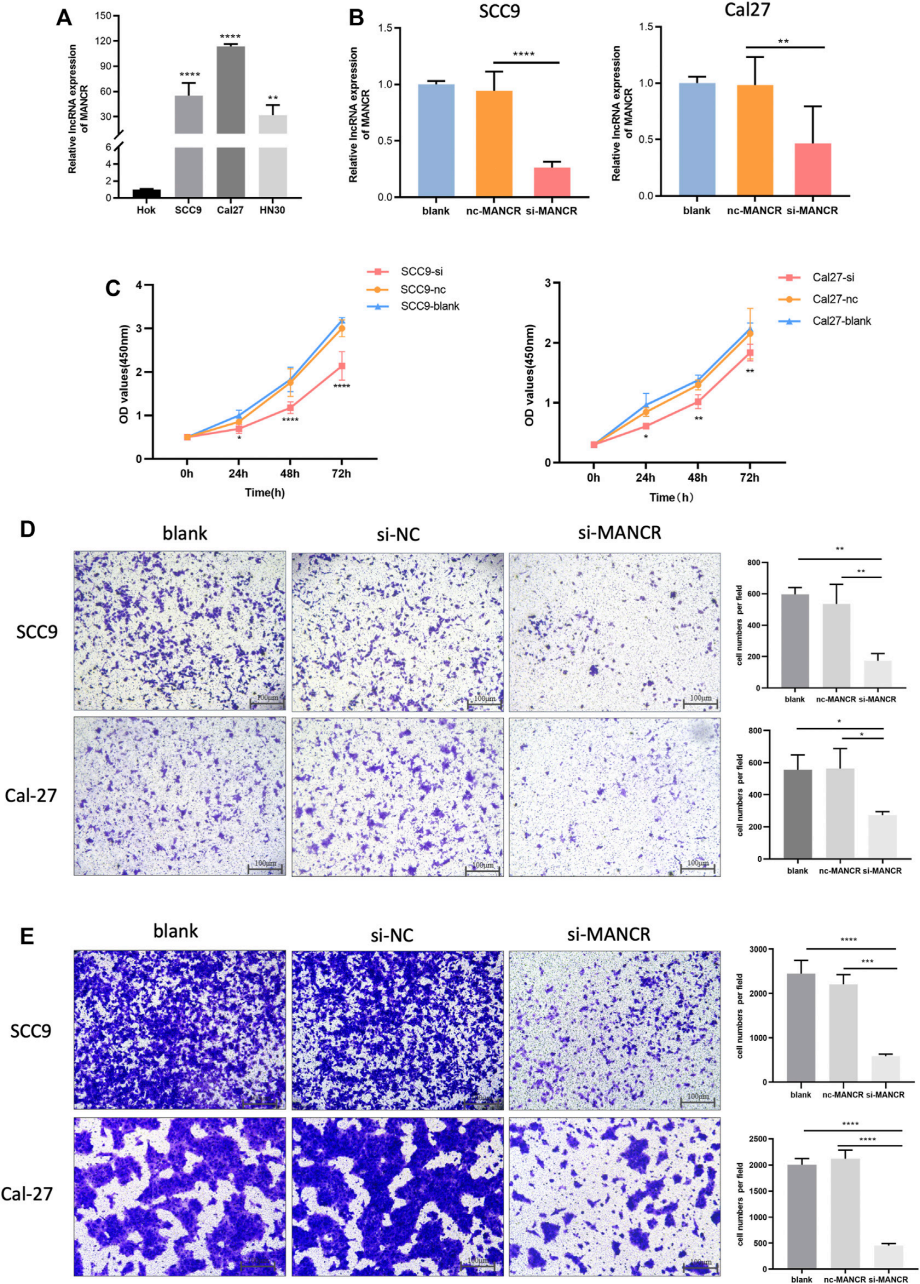
Biological effect of MANCR on HNSCC cells. **(A)** Expression of MANCR in SCC9, Cal27, HN30, and Hok cells. **(B)** Knockdown efficiency was evaluated by RT-qPCR. **(C)** Proliferation of Cal27 and SCC9 cells was examined by the CCK-8 assay. **(D)** Transwell assays were performed and indicated the invasion ability of Cal27 and SCC9 cells. **(E)** Migration ability of Cal27 and SCC9 cells were also examined by the transwell assay (*p < 0.05, **p < 0.01, ***p < 0.001, and ****p < 0.0001).

Finally, we further evaluated and validated MANCR expression in different HNSCC cell lines, and the RT-qPCR result showed that MANCR was highly expressed in Cal27, SCC9, and HN30 cell lines compared with Hok cells (Figure 7A). After that, we used the lncRNA Smart Silencer to knock down the cellular level of MANCR in Cal27 and SCC9 cell lines. The knockdown efficacy was confirmed by RT-qPCR, and we observed that MANCR expression levels in Cal27 and SCC9 cells were markedly repressed after transfection compared with the negative control (NC) and blank group (Figure 7B). CCK-8 assay showed that the knockdown of MANCR significantly inhibited the proliferation ability in both SCC9 and Cal27 (Figure 6C). The transwell assay was performed to verify that the migration and invasion ability of Cal27 and SCC9 cells were markedly decreased after MANCR knockdown (Figure 6D). Therefore, our findings indicated that MANCR may act as an oncogene and enhance HNSCC malignant progression.

## Discussion

Previous studies have demonstrated that MANCR is highly expressed in gastric cancer and breast cancer and correlated with poor patient prognosis (Tracy et al., 2018; Yao et al., 2019). However, the expression pattern of MANCR in HNSCC patients and its prognostic values are still elusive. Here, by data mining analysis from TCGA and the GTEx database, the upregulation of MANCR was observed in 17 types of tumor tissues compared with adjacent normal tissues, including HNSCC. Next, the analysis of the ROC curve showed that the AUC of MANCR was more than 0.816, indicating its potential role in diagnosis. We further validated the MANCR expression level in HNSCC patients by using qPCR. This observation suggested that MANCR might serve as a novel oncogene in HNSCC.

Based on the analysis through various tools, the upregulation of MANCR was found to predict poor prognosis such as low DSS and low OS. Our results also showed that HNSCC patients with higher MANCR expression were correlated with higher T stages, higher clinical stage, and shorter overall survival. In addition, univariate and multivariate Cox regression analyses proved that MANCR expression could be used as a high-risk factor of prognosis in HNSCC patients. These results support the values of MANCR as a biomarker in HNSCC prognosis.

The GO and KEGG enrichment analyses were extensively associated with peripheral nerves and the extracellular matrix (ECM) for highly expressed genes, such as axonogenesis, neuronal cell body, neuroactive ligand-receptor interaction, collagen-containing extracellular matrix, and ECM-receptor interaction. Existing evidence shows that these processes and structures affect the initiation, progression, therapy, and prognosis of cancers in various aspects. Researchers reported that patients with densely innervated tumors suffer from increased metastasis and poor prognosis as compared to those with less innervated tumors in HNSCC (Bakst et al., 2019; Alkhadar et al., 2020). For example, studies have reported that exosomes from HNSCC tumors could induce axonogenesis by the exosome-packaged axonal guidance molecule, ephrin B1 (Madeo et al., 2018), which could promote the metastasis and progression of cancer (Grelet et al., 2022). The change in the ECM structural constituent in the tumor microenvironment has a significant effect on the invasion and proliferation of advanced HNSCC. For instance, researchers reported that HAS3 has been shown to produce hyaluronan and subsequently contribute to HNSCC cell proliferation (Twarock et al., 2011). In addition, under the impact of the ion channel TMEM16A, the HNSCC cell was found to be closely related to the decrease in sensitivity to cisplatin and the enhancement of tumor development (Godse et al., 2017). The aforementioned results showed that MANCR is closely related to malignant cell behavior. Further analyses verified that MANCR knockdown suppressed HNSCC cell proliferation, invasion, and migration. These results suggest that MANCR may play a crucial role in the occurrence and development of HNSCC; hence, it is reasonable to speculate that MANCR may be a promising therapeutic target for HNSCC.

Immune cells are important subsets of the tumor microenvironment (TME) and it may be possible, through crosstalk with tumor cells of HNSCC, to induce immune evasion and immune surveillance (Elmusrati et al., 2021). Many studies have shown that tumor immune cell infiltration can affect the prognosis of cancer patients (Li et al., 2016; Sokratous et al., 2017). Further GSEA suggested that the genes of high MANCR expression were mainly enriched in lymphocyte-mediated immunity for HNSCC. To gain further insight into the mechanism of MANCR in HNSCC development, we performed the correlation between MANCR expression and immune cell infiltration. Our work indicated that the transcription levels of MANCR were closely correlated with various levels of immune infiltration in HNSCC. There is a significantly positive relationship between MANCR expression levels and infiltration levels of tgd cells, neutrophils, and Th1 cells, whereas negative correlations between the infiltration levels of B cells, CD8^+^ T cells, and T cells. Numerous studies have confirmed that tgd cells within tumors possess high cytotoxic activity in cancer and are related to improvement outcomes (Paul and Lal, 2016). However, the functions and prognostic role of tumor-infiltrating tgd cells in patients with HNSCC are controversial. Lu et al. (2020)reported that a high abundance of intratumoral tgd cells favors better prognosis in HNSCC. In contrast, Bas et al. (2006) reported that patients with HNSCC had a significantly increased proportion of tgd cells, particularly in patients with recurrent or metachronous second primary HNSCC, implying their tumor promoter roles in HNSCC tumorigenesis Bas et al. (2006). Consistently, our study revealed that high MANCR expression is positively correlated with tgd cells. It is hypothesized that the positive effect of the tgd cell may be partially attributed to its ability to induce an immunosuppressive tumor microenvironment and inhibit antitumor adaptive T cell immunity, which may promote tumor progression (Fleming et al., 2017). In addition, a high density of neutrophils was found in tumor tissues and cooperated with pro-tumor response and the level was associated with poor survival time in HNSCC (Li et al., 2019). In contrast to the immune surveillance role of lymphocytes in the TME, other immune cells may promote tumor growth and metastasis by favoring the TME. Tumor-infiltrating B cells in HNSCC have the potential to contribute to antitumor immunity in many ways including presenting tumor antigens to CD4^+^ T cells which were correlated with increased survival (Ruffin et al., 2021). The greater accumulation of CD8^+^ T lymphocytes in the TME was generally associated with an improved prognosis for HNSCC (Fridman et al., 2012; Nguyen et al., 2016). Taken together, our findings indicated that MANCR might be involved in the regulation of HNSCC immunity. Therefore, comprehensive studies on the association of tumor-infiltrating immune cells, immunomodulators, and MANCR in HNSCC are needed.

## Conclusions

Overall, our research exhibited that MANCR was elevated in HNSCC and it promoted the malignant progression of HNSCC. It may serve as a potential biomarker in prognostic implications for HNSCC patients. The positive correlation between MANCR and immune infiltration cells may provide novel therapeutic targets and personalized immune-based cancer therapy for HNSCC.

## Data Availability

The original contributions presented in the study are included in the article/Supplementary Materials; further inquiries can be directed to the corresponding author.
